# Low Temperature Atomic Layer Deposition of (00*l*)‐Oriented Elemental Bismuth

**DOI:** 10.1002/anie.202422578

**Published:** 2025-02-14

**Authors:** Jorge Luis Vazquez‐Arce, Alessio Amoroso, Nicolas Perez, Jaroslav Charvot, Dominik Naglav‐Hansen, Panpan Zhao, Jun Yang, Sebastian Lehmann, Angelika Wrzesińska‐Lashkova, Fabian Pieck, Ralf Tonner‐Zech, Filip Bureš, Annalisa Acquesta, Yana Vaynzof, Anjana Devi, Kornelius Nielsch, Amin Bahrami

**Affiliations:** ^1^ Leibniz-Institute for Solid State and Materials Research Dresden Helmholtzstraße 20 01069 Dresden Germany; ^2^ Department of Chemical Engineering Materials and Industrial Production University of Napoli Federico II Piazzale Tecchio 80 80125 Napoli Italy; ^3^ Institute of Organic Chemistry and Technology Faculty of Chemical Technology University of Pardubice Pardubice 53210 Czech Republic; ^4^ Inorganic Materials Chemistry Ruhr-University Bochum Universitätsstraße 150 44801 Bochum Germany; ^5^ Institute of Materials Science Technische Universität Dresden Helmholtzstraße 7 01062 Dresden Germany; ^6^ Chair for Emerging Electronic Technologies TUD Dresden University of Technology Nöthnitzer Str. 61 01187 Dresden Germany; ^7^ Wilhelm-Ostwald-Institut für Physikalische und Theoretische Chemie Leipzig University Linnéstr. 2 04103 Leipzig Germany

**Keywords:** Atomic Layer Deposition, Hall resistance, Preferential growth, Surface Chemistry

## Abstract

This study presents the first successful demonstration of growing elemental bismuth (Bi) thin films via thermal atomic layer deposition (ALD) using Bi(NMe_2_)_3_ as the precursor and Sb(SiMe_3_)_3_ as the co‐reactant. The films were deposited at a relatively low temperature of 100 °C, with a growth per cycle (GPC) of 0.31–0.34 Å/cycle. Island formation marked the initial growth stages, with surface coverage reaching around 80 % after 1000 cycles and full coverage between 2000 and 2500 cycles. Morphological analysis revealed that the Bi grains expanded and became more defined as the number of ALD cycles increased. This coalescence is further supported by X‐ray diffraction (XRD) patterns, which show a preferential shift in growth orientation from the (012) plane to the (003) plane as the film thickness increases. X‐ray photoemission spectroscopy (XPS) confirmed the presence of metallic Bi with minimal surface oxidation. Temperature‐dependent sheet resistance measurements highlight the semimetallic nature of Bi, with a room temperature resistivity of ≈200 μΩcm for the 2500 cycles Bi. Temperature‐dependent sheet resistance was also associated with a transition in carrier‐type dominance from holes at higher temperatures to electrones at lower temperatures.

## Graphical abstract

The first successful growth of elemental bismuth (Bi) thin films via thermal atomic layer deposition (ALD) using Bi(NMe_2_)_3_ and Sb(SiMe_3_)_3_ at 100 °C, achieving a GPC of 0.31–0.34 Å/cycle, is demonstrated. Films transitioned from island to full coverage, with a preferential shift in growth orientation from the (012) to the (003) plane. Metallic Bi was confirmed by XPS and TEM. The 2500‐cycle film exhibited semimetallic behavior with a resistivity of ≈200 μΩcm.

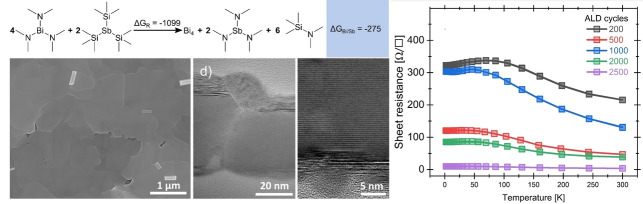

## 1 Introduction

As electronic devices shrink in size, there is an increasing demand for new material growth techniques capable of addressing the challenges of nanofabrication devices in more complex geometries.^[1]^ Traditional deposition methods, such as chemical vapor deposition (CVD) and its variants, as well as physical vapor deposition (PVD), are expected to struggle to provide the conformal coverage required for high‐aspect‐ratio structures in the most advanced nanoscale devices in the next 5 to 10 years.^[2]^

Atomic Layer Deposition (ALD) is a promising method for atomic‐level processing because it can deposit materials conformally on substrates with complex topographies.[^3^, ^4^] To fabricate these advanced devices, ALD must be able to synthesize a wide range of materials, including oxides, nitrides, and metals.^[5]^ Metals, in particular, play a critical role in the functionality of electronic devices by serving as conductors, electrodes, and interconnects.^[5]^ However, despite its potential, the range of metals that ALD can deposit requires further expansion. This stems from the complexities of finding suitable metal precursors and co‐reactants to produce high‐purity films with the desired properties and the need to control variables such as deposition temperature, precursor reactivity, and film uniformity.[^6^, ^7^]

Compounds containing group 15 elements from the periodic table have garnered particular interest in ALD research because of their appealing properties and promising applications, including thermoelectric materials, phase‐change memory devices, and photovoltaic absorbers.[^8^, ^9^] To date, antimony (Sb) is the only group 15 element that has been successfully deposited in its metallic form using ALD.[^10^, ^11^, ^12^] Although several bismuth compounds have been deposited via ALD, such as Bi_2_O_3_, Bi_2_S_3_, and Bi_2_Te_3_, metallic Bi synthesized by thermal ALD has yet to be reported. Bi(OCMe_2_^i^Pr)_3_ and H_2_O^[13]^ have been employed to deposit Bi_2_O_3_ thin films at growth temperatures ranging from 90 to 270 °C, using precursor temperatures between 85–110 °C. This process enables the formation of high‐density *β*‐phase Bi_2_O_3_ films at a growth rate of approximately 0.39 Å/cycle. On the other hand, polycrystalline Bi_2_S_3_ ^[14]^ has been deposited at 200 °C using Bi(thd)_3_ at 95 °C and H_2_S as the co‐reactant, achieving a growth rate of 0.34–0.37 Å/cycle. Rusek et al. synthesized Bi_2_Te_3_ films at a deposition temperature of 170 °C using [(Me_3_Si)_2_NBi‐μ‐NSiMe_3_]_2_ and (SiMe_3_)_2_Te as co‐reactant, with growth rates ranging from 0.66 to 3.5 Å/cycle, depending on the type of substrate.^[15]^

Bismuth, a notable semimetal within group 15,[^16^, ^17^] stands out due to its unique electrical transport properties, which make it highly suitable for various advanced technological applications such as topological insulators and thermoelectrics.[^18^, ^19^, ^20^]

One of the main challenges in fabricating thin Bi films lies in controlling both their morphology and crystal orientation. Various deposition methods, such as Sputtering and molecular beam epitaxy (MBE), have demonstrated the ability to promote specific crystallographic orientations and morphologies depending on factors like deposition energy, substrate temperature, and growth rate. For example, Kumari et al.^[21]^ studied Bi films deposited by thermal evaporation and observed that both grain orientation and size depend on film thickness and deposition temperature. At low temperatures, the films display a preferred orientation along the (00*l*) axis, forming large grains that enable continuous films after reaching a certain thickness. In contrast, at higher temperatures, such as 200 °C, Bi tends to grow with a (012) orientation and random grain sizes, which hinders the formation of a continuous layer. Similarly, Pilidi et al.^[22]^ reported that the ratio between the (00l) and (012) orientations gradually varies with deposition temperature.

In the study by Hirayama^[23]^ on Bi(111) films grown by MBE, initial Bi islands on Si(111) substrates first adopt a (110) orientation, which transitions to a (111) orientation once a critical thickness is reached. Additionally, as shown by Ushioda et al.,^[24]^ the transition in crystallographic orientation and film continuity also depends on the growth rate in PVD experiments. Low deposition rates, such as 0.035 monolayers/min, result in Bi islands that grow in the (110) orientation, remaining discontinuous and inhomogeneous. As the growth rate increases, Bi(110) islands nucleate at uniform heights and expand laterally to cover nearly the entire surface, eventually reorganizing into a more stable Bi(111) structure. The significance of this lies in the fact that the final electrical transport properties depend on the structure and morphology of the Bi films, as demonstrated in these same studies. While studies highlight the challenges in achieving uniform and oriented Bi films with various deposition techniques, ALD offers a promising alternative. Despite the successful ALD deposition of several bismuth compounds, the unique properties of elemental bismuth underscore the need for further research into its deposition via thermal ALD, which could unlock significant technological potential in areas such as topological insulators and thermoelectrics.

In this work, we report the successful deposition of metallic bismuth thin films via thermal ALD, using Bi(NMe_2_)_3_ as the precursor and (SiMe_3_)_3_Sb as the co‐reactant. The precursor Bi(NMe_2_)_3_ has been previously used at room temperature in ALD processes for the fabrication of Bi_1_Se_1_ alloys at low deposition temperatures ranging from 80 to 120 °C.^[25]^ In addition, (SiMe_3_)_3_Sb has been employed at 70 °C to grow metallic Sb films at deposition temperatures between 80 and 150 °C.^[12]^ The study focused on understanding the growth mechanism, microstructural evolution, and electrical properties of the films. We investigated how the deposition temperature and number of cycles influenced film formation, particularly the transition from island growth to continuous film, well‐defined crystalline domains, and how these structural changes impacted the films′ electrical performance.

## 2 Results and Discussion

The first part of the results focuses on the ALD growth mechanism of bismuth thin films. The reaction mechanism for the deposition is intriguing, as both metals involved can adopt a range of oxidation states (from −3 to +3/+5). The reactions may proceed through various surface‐bound intermediates and radical steps.

To give a glimpse of the reactivity of the used precursors Bi(NMe_2_)_3_ and Sb(SiMe_3_)_3_, reaction energies for different mechanisms were calculated. We should note that these reaction energies only state whether the overall mechanism is exergonic (releasing free energy, ΔG<0 ) or endergonic (requiring free energy, ΔG>0 ). Here, strongly exergonic mechanisms can often be the most likely representation due to their thermodynamic driving force. However, computational proof of a mechanism would require a detailed knowledge of the on‐surface elemental reactions and an analysis of the resulting reaction network. As these are tedious computations, we need to neglect the surface and stick to simplified gas phase calculations for this study.

**Figure 1:**
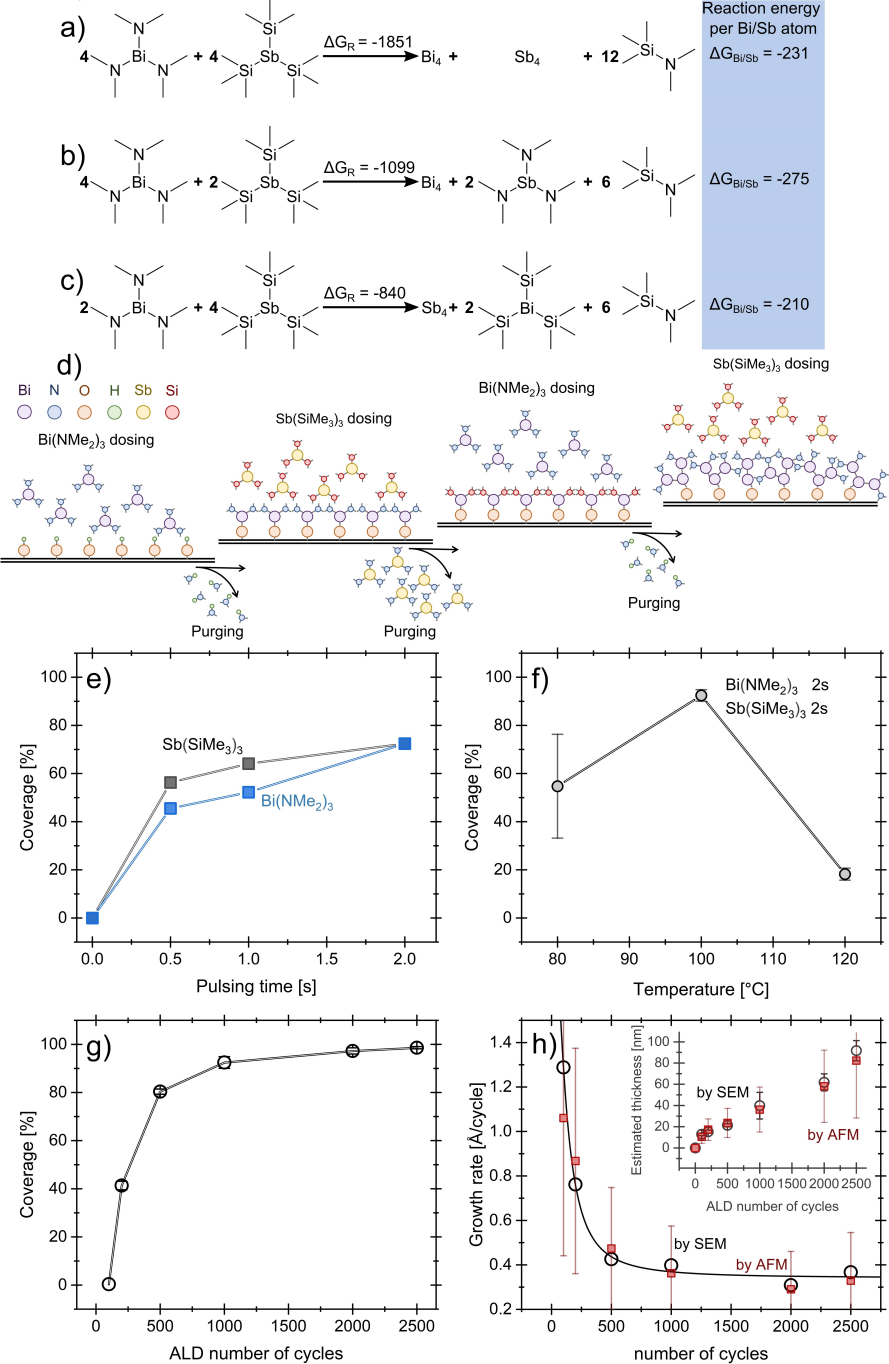
Possible gas phase reactions between Bi(NMe_2_)_3_ and Sb(SiMe_3_)_3_. In a), a direct reaction between the reactants is assumed, resulting in the simultaneous deposition of Bi and Sb. In b), a ligand exchange reaction is assumed, leading to the deposition of only Bi and the desorption of a Sb(NMe_2_)_3_ species. In c), the reverse ligand exchange is assumed with the deposition of Sb and the desorption of Bi(SiMe_3_)_3_. Reaction energies (ΔG_R_) and reaction energies per deposited Bi/Sb atom (ΔG_Bi/Sb_) at 100 °C and 10^−3^ atm are given in kJ mol^−1^. (d) Schematic representation of elemental Bi deposition process. Surface coverage as a function of (e) dosing time and (f) growth temperature. (g) Surface coverage and (h) growth rate as functions of ALD cycle number, with the inset showing the corresponding linearity curve.

Figure 1 presents possible reactions between Bi(NMe_2_)_3_ and Sb(SiMe_3_)_3_. These are gas‐phase reactions, so a reference for Bi and Sb is necessary. Instead of using the individual atoms, we used the smallest Bi_4_ and Sb_4_ clusters, as all valence electrons are saturated within these structures. Figure 1a shows the reaction in which both precursors directly react, leading to the formation of elemental Bi+Sb and the recombination of the ligands to (NMe_2_)(SiMe_3_). With −1851 kJ mol^−1^, this reaction shows the largest exergonic reaction energy. In Figures 1b and 1c, ligand exchange reactions are assumed, leading to either the formation of elemental Bi or Sb with the ligands of the former Bi or Sb precursor, respectively. These reactions have exergonic reaction energies of −1099 kJ mol^−1^ and −840 kJ mol^−1^. As a different number of elemental Bi and Sb atoms are formed within these reactions, we also calculated the reaction energy per formed Bi/Sb atom. With −275 kJ mol^−1^, the largest energy per atom is observed for the reaction, leading to the formation of only Bi. However, similar energies are observed for the other reactions with −231 kJ mol^−1^ (Bi+Sb) and −210 kJ mol^−1^ (only Sb). Due to the step‐wise nature of material deposition during an ALD process, we value the reaction energies per deposited atom more than the total reaction energies. Consequently, our data suggest that the deposition of Bi is slightly favored over the deposition of Bi+Sb or only Sb. Still, as all reaction energies are exergonic and similar in magnitude, the thermodynamic data suggest a deposition of Bi and Sb with a higher concentration of Bi.

By considering prior calculations, Figure 1d presents a hypothesized simplified deposition pathway, starting with an initial reaction between Bi(NMe_2_)_3_ and free surface hydroxyl groups. Upon exposure to the second precursor, Sb(SiMe_3_)_3_, a ligand exchange reaction occurs, releasing the volatile by‐product (Me_2_N)_3_Sb. The Bi−Bi bond forms in a subsequent reaction with another (Me_2_N)_3_Bi molecule, accompanied by the release of volatile Me_3_Si‐NMe_2_, which is removed under vacuum. Repeating this entire ALD cycle leads to the growth of elemental Bi.

Figure 1e shows the coverage as a function of dosing time for the precursor Bi(NMe_2_)_3_ and the co‐reactant Sb(SiMe_3_)_3_ in 1000 cycles. Figure S1–S2 shows the top view scanning electron microscopy (SEM) micrographs of those Bi films. The coverage rapidly increases with the dosing time of both precursors, reaching saturation values of 70–80 % at 2 s. Non‐uniform nucleation across the substrate surface, as seen in ALD of metals such as Ru^[26]^ and Pt,^[27]^ may explain the incomplete coverage, as adsorption sites can be passivated by interactions with precursor byproducts. In this Bi ALD process, the formation of −SiMe_3_ groups as byproducts may passivate active surface sites, reducing the availability of adsorption sites for precursors, thereby limiting overall coverage and promoting island formation over continuous film growth. Additionally, it is conceivable that the Bi precursor preferentially adsorbs onto already‐formed Bi nuclei rather than directly onto the substrate.

Figure 1f shows the coverage as a function of deposition temperature, with an optimal temperature around 100 °C. The top‐view SEM images of those Bi are shown in Figure S3. Beyond this, coverage decreases significantly at 120 °C. TGA studies on the Bi(NMe_2_)_3_ molecule, conducted by Rusek et al., showed that its thermal stability remains nearly 100 % at 100 °C but drops to approximately 87 % at 120 °C due to partial thermal decomposition.^[15]^ This decomposition likely reduces the efficiency of the ALD process at reactor temperatures of 120 °C by introducing impurities or altering the precursor‘s reactivity. Another factor contributing to the observed decrease in coverage at higher temperatures is the increased surface diffusion of deposited atoms. At elevated temperatures, atoms may migrate from their initial adsorption sites, leading to agglomeration and the formation of clusters rather than a uniform distribution.^[28]^ Conversely, at 80 °C, the lower coverage can be attributed to insufficient thermal energy to activate the surface reactions necessary for efficient precursor adsorption and reaction.

Figure 1g shows the surface coverage as a function of the number of ALD cycles for Bismuth films. Various illustrative top‐view SEM images are found in the supplementary material in Figures S4–S7. The growth process shows a rapid increase in surface coverage during the initial cycles, reaching ~80 % after 1000 cycles, followed by a 100 % saturation in the 2000–2500 cycle range. This trend suggests that the nucleation and expansion of isolated islands on the substrate surface dominate the initial growth phase. As these islands grow and merge, the available surface area decreases, resulting in a slower coverage rate as the process approaches saturation.

The film thickness was measured using cross‐section SEM and atomic force microscopy (AFM). Since coverage is less than 100 % for most films, AFM allowed us to measure height differences between areas with and without material. Consequently, Figure 1h includes growth rate data derived from both SEM and AFM measurements. During the early stages, the growth rate is about 1.3 Å/cycle, which can be attributed to a surface‐enhanced growth phenomenon. In this phase, the precursor molecules readily adsorb onto the fresh substrate, leading to a rapid vertical and lateral expansion of Bi islands. As these islands coalesce and the surface becomes progressively covered, the growth rate decreases and stabilizes around ~0.31–0.34 Å/cycle. This behavior arises due to the progressive reduction of the surface area of individual islands as they grow and begin to merge, a phenomenon previously described for ALD processes involving island growth.^[29]^ In this stage, the curvature of the islands increases, reducing the available surface area for deposition. As a result, the average growth rate first reaches a local maximum before decreasing as the effective deposition area shrinks and the islands coalesce. Once a continuous film forms, the deposition transitions into a more stable layer‐by‐layer mode, where the growth is primarily driven by the reaction of precursors with existing Bi nuclei rather than the Si substrate. The inset shows the film thickness as a function of the number of ALD cycles.

**Figure 2:**
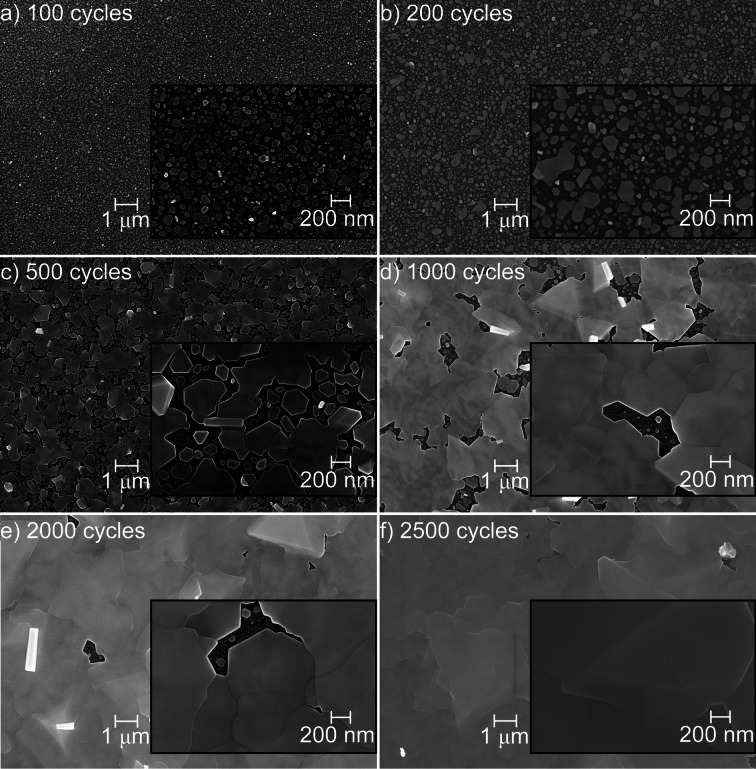
Field emission SEM images of the morphology of the Bi films deposited using Bi(NMe_2_)_3_ and Sb(SiMe_3_)_3_ at 100 °C as a function of the number of ALD cycles.

Figure 2 illustrates SEM images of the evolution of surface morphology in Bi films as a function of the ALD cycles. These micrographs were also used to determine the parameters shown in Figures 1(e–h) and are complementary to those that appear in the Supporting Information. In the early stages (Figure 2a, 100 cycles), the surface is dominated by many small, isolated nuclei. As cycles increase to 200 (Figure 2b), these nuclei grow and coalesce, forming larger islands. The coalescence process has progressed significantly by 500 and 1000 cycles (Figure 2c–d). Still, even after 2000 ALD cycles (Figure 2e), the surface becomes nearly continuous, with well‐formed grains that coalesce into an almost completely closed film after 2500 cycles (Figure 2f).

At this stage, the bismuth grains display irregular polygonal shapes that follow preferential growth directions in the crystal lattice (see X‐ray diffraction (XRD) section). In the insets, the higher resolution reveals the polygonal shapes of the bismuth grains more clearly, as well as how these grains grow and merge as the number of ALD cycles increases. By 1000 cycles, the grain boundaries become more pronounced as the grains grow and merge. The trend continues at 2000 and 2500 cycles, when the grains have merged into a continuous film of smooth grains, and the grain boundaries become even more well‐defined.

**Figure 3:**
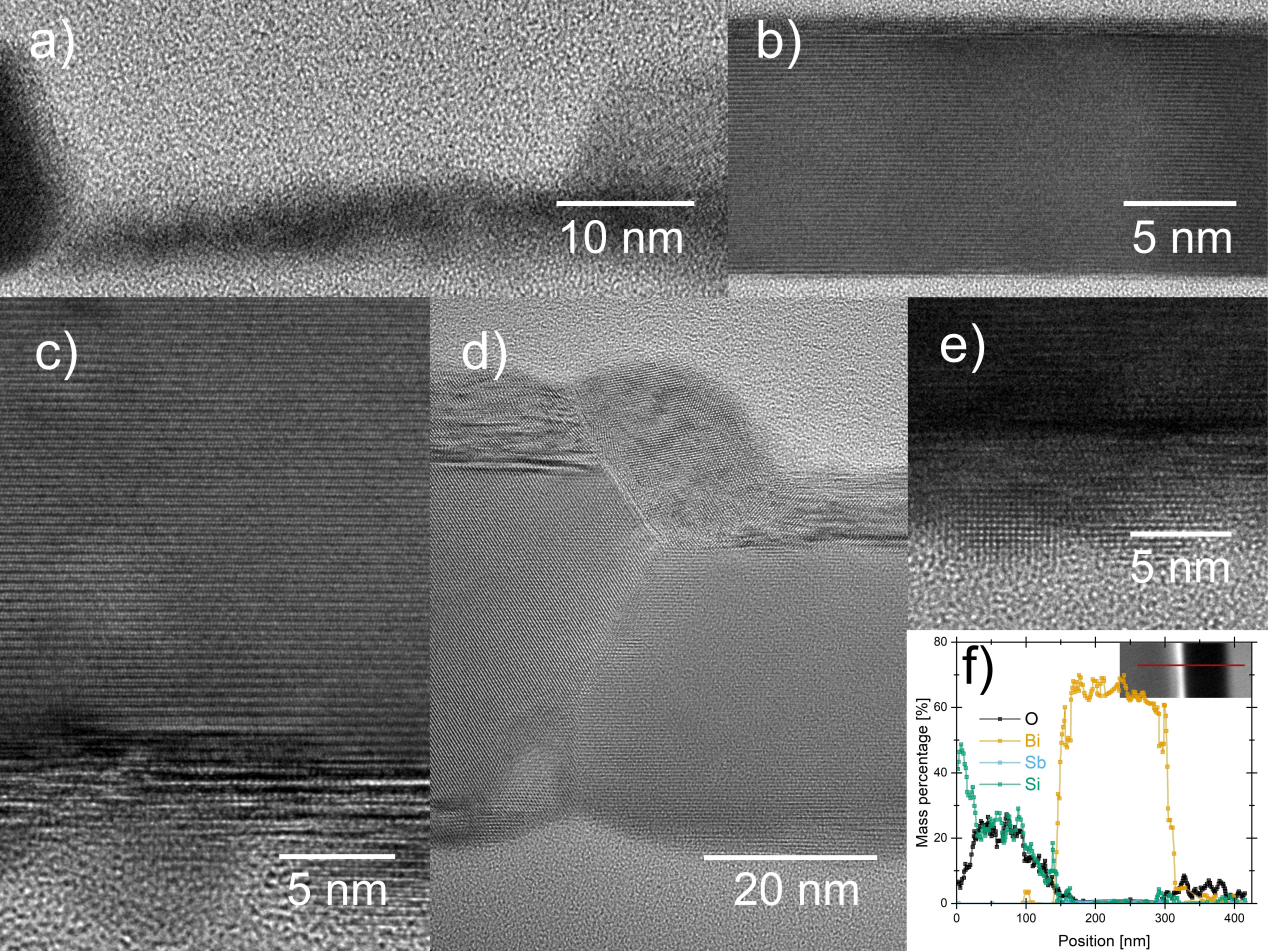
Cross‐section TEM images of Bi films deposited using Bi(NMe_2_)_3_ and Sb(SiMe_3_)_3_ at 100 °C. (a) Bi clusters formed after 500 ALD cycles, linking larger crystalline Bi islands (see also Figure S8a–b); (b) high‐magnification image showing a well‐crystallized Bi film; (c) interface transitioning from defective to highly crystalline Bi with c‐axis preferential orientation; (d) highly defective termination layer on top of the well‐ordered Bi crystals, with varying thickness (3–30 nm); (e) small clusters at the SiO_2_/Bi interface, which can crystallize in various orientations; and (f) EDX line scan profile, measured across the film from the surface to the Si/SiO_2_ substrate, showing the elemental distribution of Bi, O, Sb, and Si. The inset highlights the scanned region in the TEM image.

Cross‐section transmission electron microscopy (TEM) images provide further insight into the growth of the Bi layers, as shown in Figure 3. After 500 cycles, not completely crystalline Bi clusters (Figure 3a and Figure S8a–b) of a few nm in size link the otherwise well‐crystallized Bi islands (Figure 3b). As the number of cycles increases, the initially amorphous clusters crystallize and allow the growth of a closed Bi layer on top (Figure 3c). Initially that closed Bi layer is defective, but after about 5 nm in thickness the crystalline order increases and develops for the major part c‐axis preferential orientation (Figure 3c). The large Bi islands that grow directly on the SiO_2_ substrate develop high crystallinity after a few atomic layers (Figure 3b). A highly defective termination layer can be clearly identified on top of the well‐ordered Bi crystals (Figure 3d). The termination layer has a strongly varying thickness, from about 3 nm to 30 nm, depending on position. The small clusters at the bottom of the layer can crystallize in a range of (Figure 3e). Similarly to what can be inferred from the images shown here, thickness‐dependent crystal growth of Bi has been reported recently.^[30]^ energy‐dispersive X‐ray (EDX) spectra collected across layers of 2500 cycles show a clear separation between Bi and O from the SiO_2_ substrate (Figure 3f). The granularity of the bottom and the termination layer at the top could lie behind the slightly higher O proportion at the edges. Otherwise, the interior of the Bi layer shows no O signal beyond the noise.

**Figure 4:**
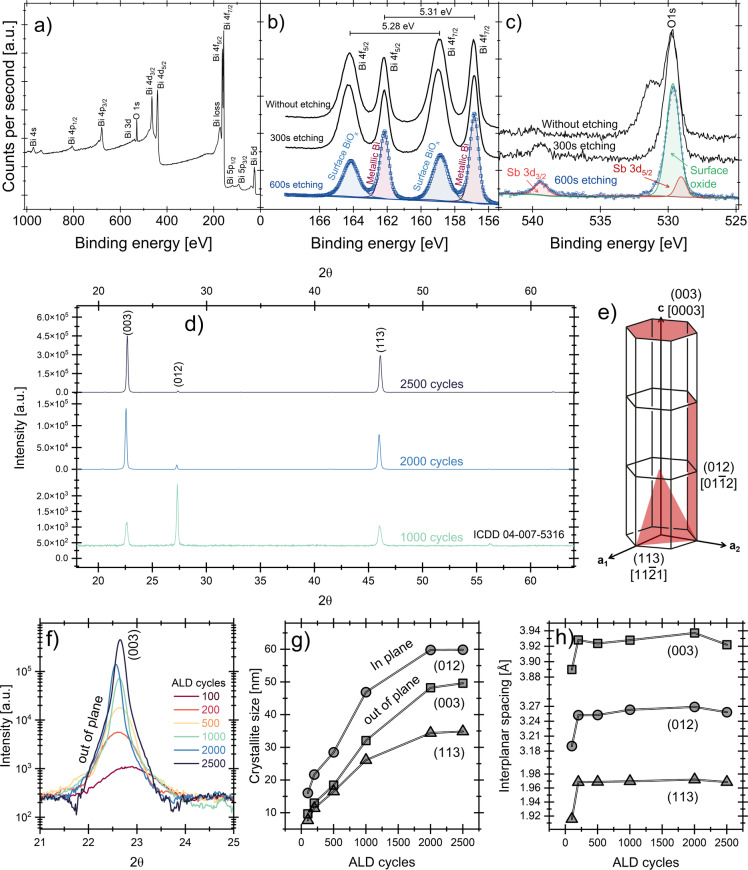
(a) Survey XPS spectrum of the Bismuth films grown at 100 °C and 2500 cycles, (b) high‐resolution XPS spectra of the Bi(4 f) signal, and (c) high‐resolution XPS spectra of the O(1s) signal. (d) XRD patterns of the Bi films corresponding to 1000, 2000, and 2500 cycles. (e) 3D schematic of the (012) and (003) plane directions. (f) Evolution of the (003) diffraction peak as a function of the number of ALD cycles. (g–h) Grain size and interplanar spacing as a function of the number of ALD cycles.

Figure 4a–c shows the surface chemical composition of the Bi films deposited on Si(100) at 100 °C and 2500 cycles, analyzed using X‐ray photoelectron spectroscopy (XPS). Figure 4a presents the survey spectrum, which shows signals for bismuth and oxygen, with no carbon contamination detected. The presence of oxygen is attributed to superficial oxidation due to atmospheric exposure during sample handling. The EDX results from TEM (Figure 3f), showed that there is no oxygen or other contaminants were detected in the bulk of the film. Ar^+^ ion etching was used, and high‐resolution Bi(4 f) spectra were measured, as shown in Figure 4b. The spectra show peaks for metallic Bi at 156.9 eV (marked in red) and Bi_2_O_3_ at 159.3 eV (marked in blue). Additionally, the Bi 4(f_7/2_)−Bi(4f_5/2_) orbital splitting is 5.31 eV for metallic Bi and 5.28 eV for Bi_2_O_3_, consistent with previously reported values.^[31]^ After 300 and 600 seconds of etching, the Bi_2_O_3_ signal appears to decrease slightly.

Figure 4c, however, shows the O(1s) signal centered at 529.8 eV before and after etching, which follows a similar pattern to the Bi(4 f) signal. After 300 and 600 seconds of etching, the signal corresponding to oxygen at approximately 531.3 eV due to oxygen from hydroxyl groups,^[32]^ diminishes. Additionally, the Sb(3d) signal is revealed at 529.2 eV and 539.64 eV, with positions and orbital splitting corresponding to Sb_2_O_3_ formed due to the film's superficial oxidation. Sb residues in the Bi films may result from incomplete reactions between the Bi(NMe_2_)_3_ and Sb(SiMe_3_)_3_ or from side reactions that produce Sb‐containing byproducts, which are then deposited alongside the bismuth. The concentration of Sb in Bi film is approximately 2.2 %. Since Sb was not detected in different areas analyzed by TEM, we can infer that this Sb contamination occurs at random sites, as the XPS spot size is 650 μm.

In the same Figure 4, the XRD analysis of Bi thin films is presented. Figure 4d shows the patterns for films grown after 1000, 2000, and 2500 ALD cycles. The peaks corresponding to the (003), (012), and (113) planes are visible and correspond to the reference ICDD card no. 04‐007‐5316. Bi film grows disordered during the initial growth phase (before 1000 cycles), with no clear preferential orientation. As the number of cycles increases to around 1000, the film undergoes preferential in‐plane growth, primarily along the (012) plane, with Bi islands growing and merging laterally ([$01\bar{1}2$ ] direction in Figure 4e). This stage corresponds to the coalescence of these islands into a more continuous structure, consistent with the surface coverage trends discussed earlier. In the final stage, between 2000 and 2500 cycles, the growth shifts towards a combination of in‐plane and out‐of‐plane growth, with the preferential orientation transitioning from the (012) plane to the (003) and (113) planes, reflecting the transition from lateral growth to vertical one.

Such behavior is typical in Bi film growth processes like thermal evaporation or pulsed laser deposition, where the combination of deposition parameters influences the growth mode. Kumari et al.^[21]^ observed that at lower temperatures (30–100 °C), the Bi films grow laminar, with grains that coalesce more uniformly across the substrate. However, as the temperature rises closer to 200 °C, the mobility of the Bi atoms increases significantly, leading to the formation of hexahedron‐like grains and a bimodal particle size distribution, reflecting the transition from laminar to columnar growth as the films become dominated by large polycrystalline grains with increased roughness. The high temperatures often also promote films that are not fully continuous due to grain segregation and island formation. Dauscher et al.^[33]^ also found that using pulsed laser deposition at 200 °C, Bi films grow in a three‐dimensional Volmer‐Weber mode during the early deposition stages. Then, as the film thickenss, these islands gradually merge into a more continuous structure. However, voids and surface roughness can persist, particularly on glass substrates, where the coalescence is less efficient. In both cases, Bi tends to crystallize in a rhombohedral structure with preferential orientations along the (012) or (003) planes, influenced by temperature and film thickness.

In addition, Figure 4f shows the evolution of the (003) plane as a function of the number of ALD cycles. The signal shifts the 2θ angle towards lower values and an increased intensity. Using the Scherrer equation, D=κλ/βcosθ ,^[34]^ the crystallite sizes for the three planes were calculated by using a k factor of 0.9, which corresponds to spherical grain shapes. It was found that the crystallite size along the (012) plane increased from 16 nm to 60 nm as the number of ALD cycles rose from 100 to 2500, while the crystallite size in the (003) direction grew from 9 nm to 50 nm and for the (113) plane, the crystallite size increased from 7 nm to 34 nm, as shown in Figure 4g. Additionally, as shown in Figure 4h, by calculating the interplanar spacing using equation 2dsinθ=nλ ,^[35]^ It was determined that the interplanar spacing increased from 3.19 Å to 3.25 Å for the (012) plane, from 3.88 Å to 3.92 Å for the (003) plane, and from 1.91 Å to 1.96 Å for the (113) plane.

Compared to ALD, the crystallographic orientation of Bi thin films obtained through various growth methods also strongly depends on growth conditions. For instance, Wang et al.^[36]^ observed that Bi films grown by MBE at 100 °C tend to have a (00*l*) orientation when their thickness is below 58 nm. However, as the thickness increases, the films reorient toward the highly crystalline (012) direction. On the contrary, Boffoué et al.^[37]^ found that Bi films deposited by pulsed laser deposition (PLD) on glass substrates at room temperature initially show a strong preference for the (012) orientation when the films are nearly continuous at around 30 nm thickness, but beyond that, the Bi reorients towards the (003) direction. Furthermore, Rodil et al.^[38]^ demonstrated that increasing the energy of the laser used for PLD can shift the preferred orientation of Bi films from the (003) plane to the (012) plane.

**Figure 5:**
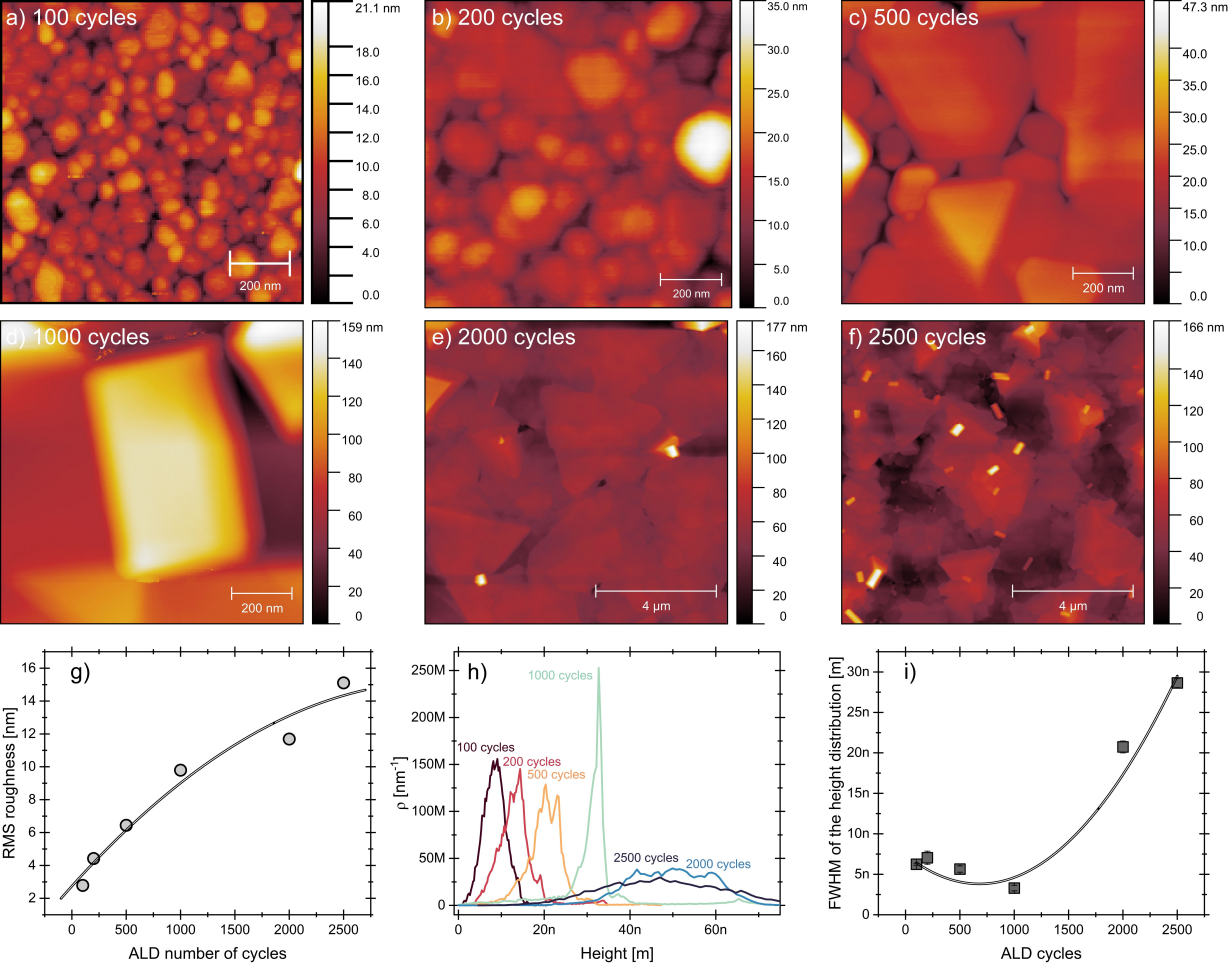
AFM analysis of the surface morphology of Bi films deposited by ALD at 100 °C. (a–f) AFM images for 100, 200, 500, 1000, 2000, and 2500 ALD cycles. (g) RMS roughness as a function of the ALD cycles. (h) Height distribution profiles of the AFM measurements. (i) FWHM of the height distribution as a function of the ALD cycles.

AFM was used to examine the surface topology of Bi films, providing a more detailed analysis than SEM, from initial nucleation to forming a continuous and textured film. Figure 5a–f presents the AFM images for the Bi films of 100, 200, 500, 1000, 2000, and 2500 ALD cycles. Additional AFM images are provided in Figure S10. During the early stages of deposition (100–200 cycles), the surface is characterized by small, densely packed grains that start to merge and expand. Beyond 500 ALD cycles, Bi grains are larger and more defined, and irregular polygonal shapes emerge, forming distinct features such as triangles. As the bismuth film becomes continuous at 2000 and 2500 cycles, the polygonal shape grains stack and coalesce into larger structures, growing vertically and laterally. This transition is further supported by an increase in surface roughness, as shown in Figure 5g, where the root mean square (RMS) values consistently rise from 2.8 to 15.1 nm with the number of ALD cycles.

Furthermore, Figure 5h presents the height distribution profiles obtained from AFM measurements, revealing a significant increase in peak height with the number of cycles. Another critical observation is the trend in the full width at half maximum (FWHM) of the height distribution, as shown in Figure 5i. Between 100 and 1000 cycles, the FWHM narrows, reflecting a period when the polygonal shapes become more defined, and the grains grow more uniformly. However, as the cycle number increases to 2000 and 2500, the FWHM broadens again. The film transitions from lateral growth to grain stacking at these stages, resulting in a more textured surface. This stacking introduces more height variation across the film, reducing the homogeneity seen during earlier growth phases.

**Figure 6:**
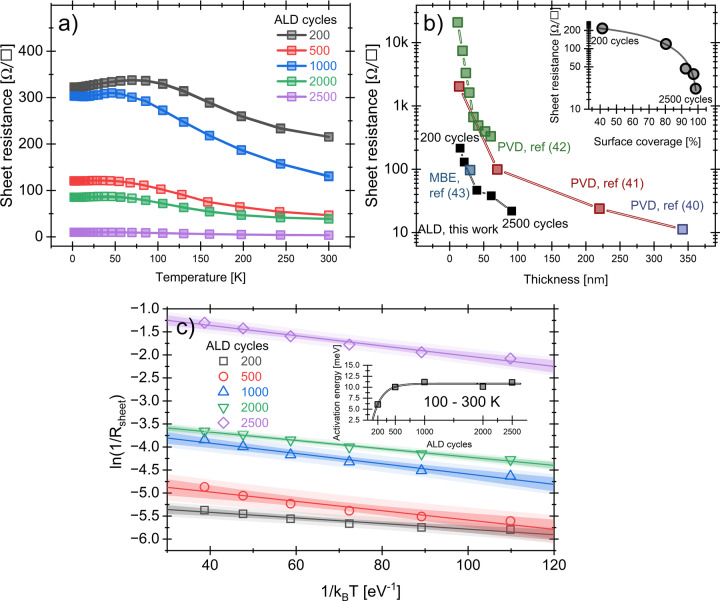
(a) Temperature dependence of the sheet resistance of the Bi films from 300 K to 2 K, and (b) 300 K sheet resistance of Bi films as a function of thickness with an inset showing the surface coverage dependence. (c) Arrhenius plot of the $ln1/R_{sheet}$ vs $1/k_{B}T$ for Bi‐ALD Bi films grown by ALD with 200, 500, 1000, 2000, and 2500 cycles between 100 and 300 K. The inset shows the activation energy values as a function of film thickness.

Finally, Figure 6a illustrates the temperature dependence of the sheet resistance of the Bi films from 2 to 300 K. The shape of the curves for all curves is similar; as the temperature decreases, the sheet resistance increases until it stabilizes and decreases slightly at temperatures below 100 K. At room temperature, the sheet resistance for the 2500 cycles was 3.6 Ω/□, corresponding to a resistivity of approximately 200 μΩcm, which is close to the 130 μΩcm reported for bulk Bi.^[39]^ Then, as the temperature decreases from 300 K to lower temperatures, the sheet resistance increases to 9.9 Ω/□. Moreover, between 35 K and 2 K, it reduces slightly. This behavior reflects the semimetallic nature of bismuth. It can be attributed to a combination of factors previously observed in Bi films, such as a drop in the carrier concentration and mobility, as well as a reduction of the electron‐phonon scattering.^[40]^

On the other hand, Figure 6b presents the sheet resistance of Bi films at 300 K as a function of nominal thickness, comparing our results with reference data from films fabricated by other techniques, such as PVD[^40^, ^41^, ^42^] and MBE.^[43]^ As observed, resistivity increases significantly as the film thickness decreases below 100 nm. This can be attributed, for example, to Quantum Size Effects, which become particularly pronounced when the film thickness approaches the mean free path of charge carriers in Bi, as discussed by Duggal and Rup.^[44]^ The inset in Figure 5b shows the sheet resistance as a function of surface coverage, as previously described. As the surface coverage increases from 40 % (Bi, 200 cycles) to nearly 100 % (Bi, 2500 cycles), the sheet resistance decreases by approximately a factor of 10, reflecting the transition from a discontinuous film composed of isolated islands to a continuous film. The 100‐cycle film did not exhibit electrical continuity, and therefore, it was not included in the sheet resistance measurements. The largely amorphous clusters linking Bi islands shown in Figure 3a and Figure S8 create a conducting path between crystalline islands. Figure 6c shows the Arrhenius plot of $\ln1/R_{sheet}$ vs $1/k_{B}T$ for Bi‐ALD films of different cycles between 100 and 300 K. It was found that the activation energy tends to stabilize at approximately 11 meV as the number of ALD cycles increases, aligning with previously reported values.^[45]^

**Figure 7:**
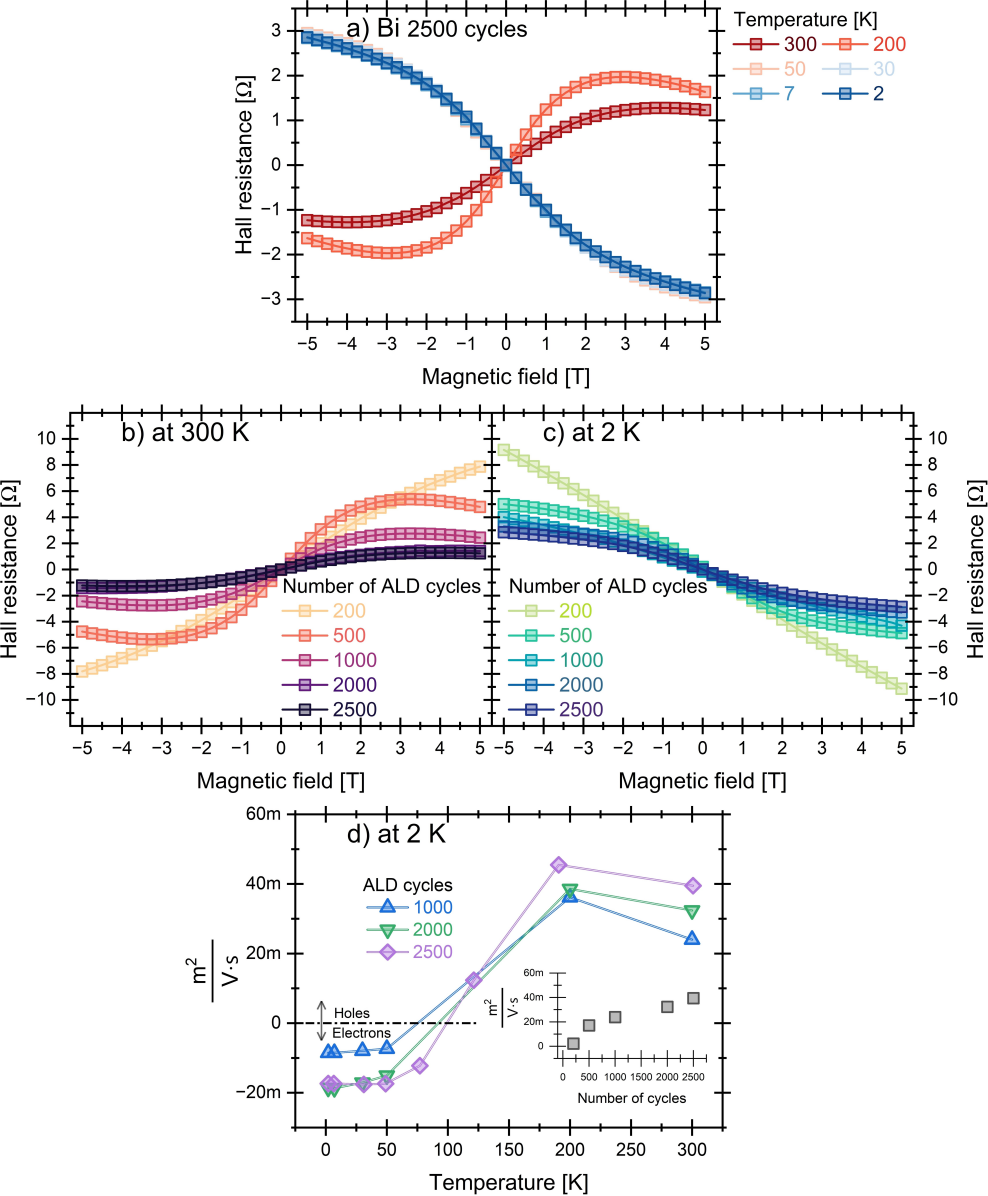
(a–b) Hall resistance of Bi films deposited by 2500 cycles as a function of the applied magnetic field, measured at various temperatures from 300 to 2 K. (b–c) Hall resistance at 300 K and 2 K for Bi films of varying thicknesses (200 to 2500 cycles). (d) Temperature dependence of mobility‐like parameter for Bi films grown by ALD.

Figure 7a displays the Hall resistance R_H_ of the Bi film of 2500 cycles as a function of the applied magnetic field at various temperatures from 300 K to 2 K. At higher temperatures, such as 300 K, 200 K, and 50 K, the Hall resistance changes from negative to positive as the magnetic field increases, indicating dominant hole‐type carriers. In contrast, at low temperatures, 30, 7, and 2 K, the values shift from positive to negative with increasing magnetic field, suggesting a dominance of electron‐type carriers, which is consistent with previous observations.^[46]^ It is generally accepted that the effective masses of electrons and holes in bismuth are sufficiently different^[47]^ to influence their behavior as the temperature decreases. As a result, both carrier types contribute to electrical conduction, with one type potentially dominating over the other depending on temperature‐dependent factors like scattering by phonons or crystal defects.[^48^, ^49^]

Furthermore, Figures 7b–c further show the Hall resistance at 300 K and 2 K, respectively, for ALD Bi films with varying thicknesses. In both cases, the Hall resistance decreases as the film thickness increases. In the particular case of elemental Bi, two‐ or three‐band models^[39]^ were employed in recent literature[^50^, ^51^, ^52^] in order to accurately determine parameters such as mobility and charge carrier concentrations, accounting for the fact that holes and electrons contribute relevantly to transport.

Nonetheless, to understand the basic trends in our materials, we will perform a simplified Hall‐mobility analysis based on the Hall coefficient $C_{H}$ in the range ±1 T divided by the sheet resistance. This will allow for a more relevant comparison with previous results. Figure 7d shows the temperature dependence of the Hall mobility obtained for Bi films with better continuity of 1000, 2000, and 2500 cycles. The transition in carrier type dominance is seen as temperature reduces, with electron‐dominated conduction at lower temperatures (T<100 K), where all the samples exhibit negative mobility values. As the temperature rises (T>150 K), the mobility value becomes positive, signifying a transition to the hole‐dominated transport, as discussed above. The obtained mobility‐like values at 300 K increase as the number of cycles increases, as shown in the inset in Figure 7d, with an equivalent mobility value of 400 cm^2^/V⋅s for the 2500‐cycle ALD Bi film at 300 K. Additionally, the corresponding charge carrier concentration for the Bi film grown with 2500 cycles was estimated to be around 10^20^ cm^−3^.

## 3 Conclusions

A new ALD process was developed at temperatures as low as 100 °C, utilizing Bi(NMe_2_)_3_ and Sb(SiMe_3_)_3_ as precursors. Bismuth thin films were successfully deposited, starting with a rapid growth rate of 1.3 Å/cycle during the initial stages, stabilizing to 0.31–0.34 Å/cycle. Preliminary computational investigations suggest a thermodynamic driving force for Bi deposition over Sb deposition. The surface coverage rapidly increased during the early stages, reaching ~80 % after 1000 cycles and approaching 100 % saturation between 2000 and 2500 cycles. The films transitioned from isolated nuclei during the initial deposition stages to continuous, well‐defined grains as the number of ALD cycles increased. XRD analysis revealed a shift in preferential growth from the (012) plane in the early stages of growth to the (003) plane in the subsequent, indicating a transition from in‐plane to a combination of in‐plane and out‐of‐plane growth as the number of cycles increased. Electrical measurements indicated that the sheet resistance values decreased as the film thickness increased. For films grown by 2500 ALD cycles, the resistivity decreased to 200 μΩ⋅cm at 300 K, close to the 130 μΩ⋅cm reported for bulk Bi. Furthermore, as the temperature decreased from 300 K to 2 K, the sheet resistance of the films increased, consistent with the semimetallic nature of bismuth. Films with lower thickness exhibited increased sheet resistance due to quantum size effects and enhanced surface and grain boundary scattering. Hall effect measurements revealed that bismuth films shifted from hole‐type to electron‐type conductivity at lower temperatures, driven by the reduced electron concentration and enhanced hole mobility caused by quantum confinement effects and the semimetal‐to‐semiconductor transition in thinner films.

## Conflict of Interests

The authors declare no conflict of interest.

## Supporting information

As a service to our authors and readers, this journal provides supporting information supplied by the authors. Such materials are peer reviewed and may be re‐organized for online delivery, but are not copy‐edited or typeset. Technical support issues arising from supporting information (other than missing files) should be addressed to the authors.

Supporting Information
