# Supplementary material for: Low Temperature Atomic Layer Deposition of (00l)‐Oriented Elemental Bismuth
Source: Angew Chem Int Ed Engl. 2025 Feb 14;64(15):e202422578. doi: 10.1002/anie.202422578 (PMC11976198; doi:10.1002/anie.202422578)
Supplement: Supplementary file 1 — Supporting Information [file ANIE-64-e202422578-s001.pdf]

Angewandte Chemie
Eine Zeitschrift der Gesellschaft Deutscher Chemiker

Supporting Information

# Low Temperature Atomic Layer Deposition of (00*l*)-Oriented Elemental Bismuth

*J. L. Vazquez-Arce, A. Amoroso, N. Perez, J. Charvot, D. Naglav-Hansen, P. Zhao, J. Yang, S. Lehmann, A. Wrzesińska-Lashkova, F. Pieck, R. Tonner-Zech, F. Bureš, A. Acquesta, Y. Vaynzof, A. Devi, K. Nielsch*, A. Bahrami**

# Supporting information

# Low Temperature Atomic Layer Deposition of (00*l*)-Oriented Elemental Bismuth

Jorge Luis Vazquez-Arce[1†], Alessio Amoroso[1,2†], Nicolas Perez[1], Jaroslav Charvot[3], Dominik Naglav-Hansen[4], Panpan Zhao[1], Jun Yang[1,5], Sebastian Lehmann[1], Angelika Wrzesińska-Lashkova[1,6], Fabian Pieck[7], Ralf Tonner-Zech[7], Filip Bureš[3], Annalisa Acquesta[2], Yana Vaynzof[1,6], Anjana Devi[1,4], Kornelius Nielsch[1,5*], Amin Bahrami[1*]

*1. Leibniz-Institute for Solid State and Materials Research Dresden, Helmholtzstraße 20, 01069 Dresden, Germany*

*2. Department of Chemical Engineering, Materials and Industrial Production, University of Napoli Federico II, Piazzale Tecchio 80, 80125, Napoli, Italy*

*3. Institute of Organic Chemistry and Technology, Faculty of Chemical Technology, University of Pardubice, Pardubice 53210, Czech Republic*

*4. Inorganic Materials Chemistry, Ruhr-University Bochum, Universitätsstraße 150, 44801 Bochum, Germany*

*5. Institute of Materials Science, Technische Universität Dresden, Helmholtzstraße 7, 01062 Dresden, Germany*

*6. Chair for Emerging Electronic Technologies, TUD Dresden University of Technology, Nöthnitzer Str. 61, 01187 Dresden, Germany*

*7. Wilhelm-Ostwald-Institut für Physikalische und Theoretische Chemie, Leipzig University, Linnéstr. 2, 04103 Leipzig, Germany*

# Description

The supplementary file provides the experimental part and complementary data on the morphological and structural evolution of Bi thin films grown by ALD. The figures include SEM and TEM micrographs of different numbers of cycles, pulsing times, and deposition temperatures. The document also includes AFM images of the same samples are also shown.

**[†] Contributed equally**
*** Corresponding authors: Amin Bahrami (a.bahrami@ifw-dresden.de), Kornelius Nielsch (k.nielsch@ifw-dresden.de)**

# 1 Experimental Procedure

## 1.1 ALD Process

The Bi films were grown in a thermal ALD reactor (Veeco, Savannah S100) at 80, 100, and 120 °C on Si (100) substrates as well as Si (100) substrates with 100 nm thermally grown $SiO_2$. The Bi films grown on Si were used for thin film characterization, whereas those grown on Si/$SiO_2$ were used for electrical characterization. The precursors used were $Bi(NMe_2)_3$ (prepared according to Rusek *et al.* [1]) and $Sb(SiMe_3)_3$ kept at 45 and 60 °C, respectively, and the carrier gas was ultra-high purity nitrogen. High-purity $Sb(SiMe_3)_3$ is essential for this process, as previous attempts with lower-purity material were unsuccessful. To address this, we synthesized the compound using a modified procedure, yielding a highly pure product with a yield of 87%, corresponding to 44.74 g in the reaction[2], [3], [4], [5]. It's important to note that the compound's sensitivity increases with its purity. $Sb(SiMe_3)_3$ is highly sensitive to light, water, and oxygen, so it must be handled in the dark under a protective gas atmosphere. The Bi growth recipe went as follows: Bi dosing (2s) → exposure time (15s) → purging (20s) → Sb dosing (2s) → exposure time (15s) → purging (20s). Here, exposure refers to the time the precursor remains in the chamber to facilitate its reaction with the surface. During the exposure time, the working pressure reached 1-2 Torr. The experiments involved growing Bi films of different thicknesses using a number of cycles ranging from 100 to 2500.

## 1.2 Characterization

The surface morphology of the Bi films was measured using a field emission scanning electron microscope (FESEM Sigma 300-ZEISS) with an accelerating voltage of 10 kV. SEM cross-section images were taken for the thickness measurements, and the values were averaged over approximately five different regions of each sample. TEM images and EDX elemental analysis were performed in a TECNAI G20 microscope (Thermo Fisher), and high-resolution TEM images were acquired with a Titan3 80 − 300 microscope (Thermo Fisher). Surface coverage was determined from SEM images using ImageJ software by analyzing the contrast differences between areas with and without deposited material. Coverage was calculated for each sample from 3 to 5 images, and the reported error corresponds to the standard deviation of the obtained coverage values. The chemical composition of the Bi film surfaces was characterized using X-ray photoelectron spectroscopy (XPS, ESCALAB 250Xi from Thermo Scientific) with a monochromated Al Kα XR6 source (hν = 1486.6 eV) and a measurements spot size of 650 microns. Survey scans and high-resolution measurements were performed with a pass energy of 20 eV. Depth profiling was performed using an argon gas cluster ion beam with large argon clusters (Ar2000) and an energy of 4 keV generated by a MAGCIS dual-mode ion source. The etching spot size was (2.5 × 2.5) $mm^2$. The crystallinity of the Bi films was studied by X-ray

diffraction (XRD) using the Bragg-Brentano configuration with a Co Kα radiation source (D8 Advance, Bruker). The measurements were performed with a step of 0.02° in 2θ and an acquisition time of 1 second per step. Moreover, atomic force microscopy (AFM, Bruker Icon) was used to examine film topography in 1 × 1 µm and 10 × 10 µm scan areas. The tip was operated in contact mode. Finally, the electrical characterization was carried out using a Physical Property Measurement System (PPMS, Quantum Design) in a vacuum of about 1 Torr and a temperature range of 2 to 300 K. Sheet resistance was evaluated using the Vander Pauw configuration, and Magnetoresistance was measured using magnetic field sweeps ranging from -5 T to 5 T.

### 1.3 Computational methods

All calculations were performed with Orca[6], [7], [8], [9] (version 6.0). Structures were preoptimized with the PBE[10], [11] functional, while the final structures were optimized with the B3LYP[12], [13], [14], [15] functional. On this level, frequency calculations were performed to confirm the presence of minimum structures as well as to calculate the Gibbs energy correction at 100°C and $10^{-3}$ atm (760 mTorr). The D4[16], [17], [18] dispersion correction and def2-TZVPP[19], [20] basis set were used for all density functional theory calculations. The final electronic energy was derived in DLPNO-CCSD(T)[21], [22], [23], [24], [25], [26] calculations. A complete basis set extrapolation[27], [28] with the def2-TZVPP and def2-QZVPP basis set was performed here. The final Gibbs energy values were derived as a combination of the CCSD(T) electronic energies and the B3LYP Gibbs energy corrections. All calculations were performed using the standard integration grid and tight settings for the wave function and force convergence. This resulted in thresholds of 10-8 Eh for the energy convergence (wavefunction) and 10-4 Eh bohr-1 for the largest gradient (force), among other thresholds set by Orca. The resolution of identity[29], [30], [31], [32] was used where possible. Computational data are available through the Zenodo repository.

## 2 Complementary data

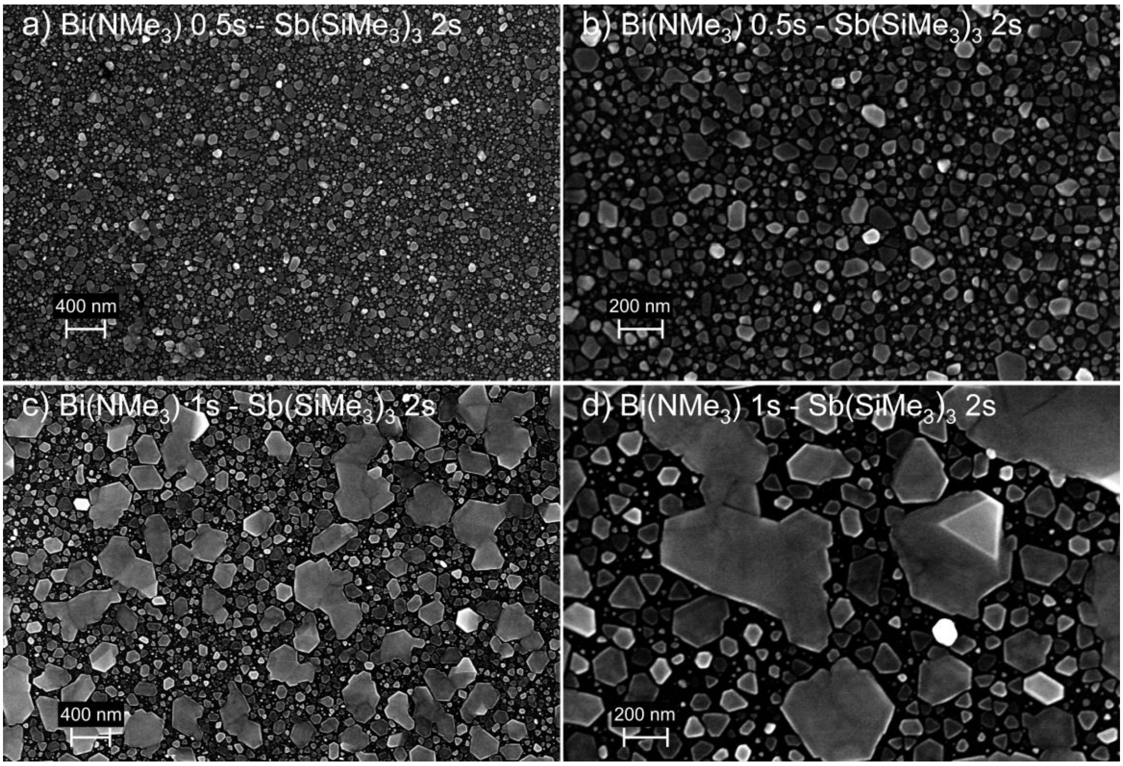


Figure S1. Top-view SEM Micrographs for 1000 ALD Cycles by varying the Bi precursor dosing while keeping the Sb one.

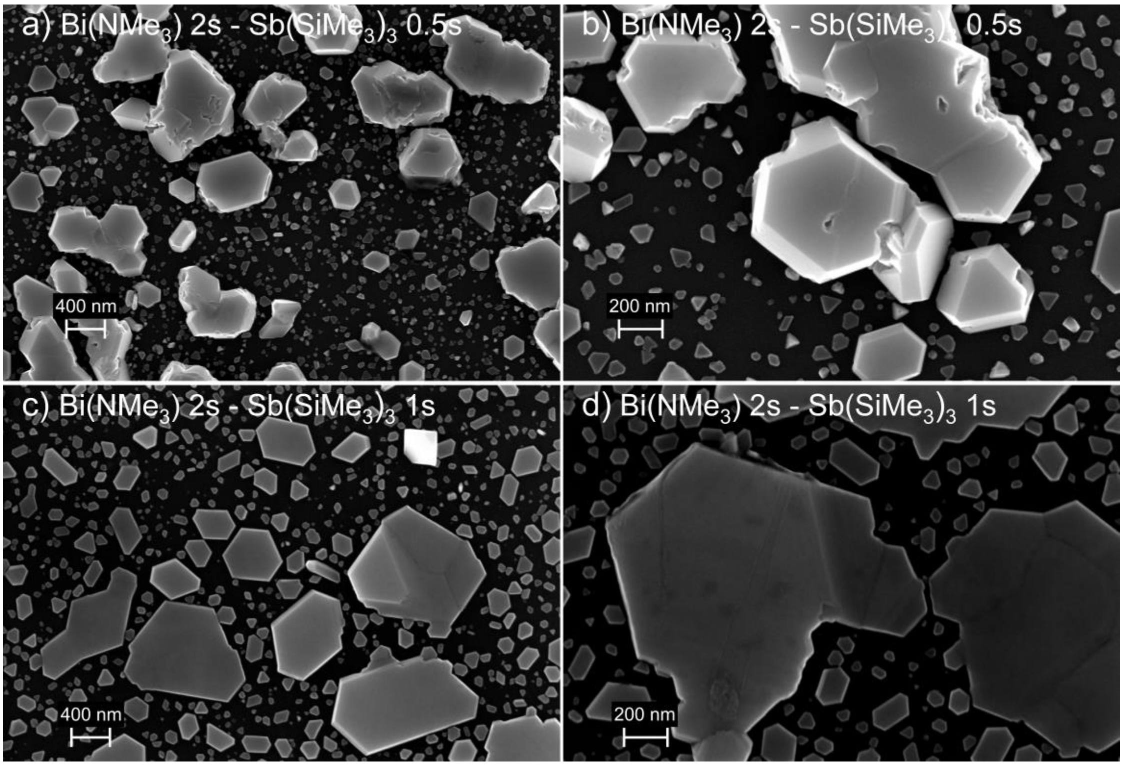


Figure S2. Top-view SEM Micrographs for 1000 ALD Cycles by varying the Sb precursor dosing while keeping the Bi one.

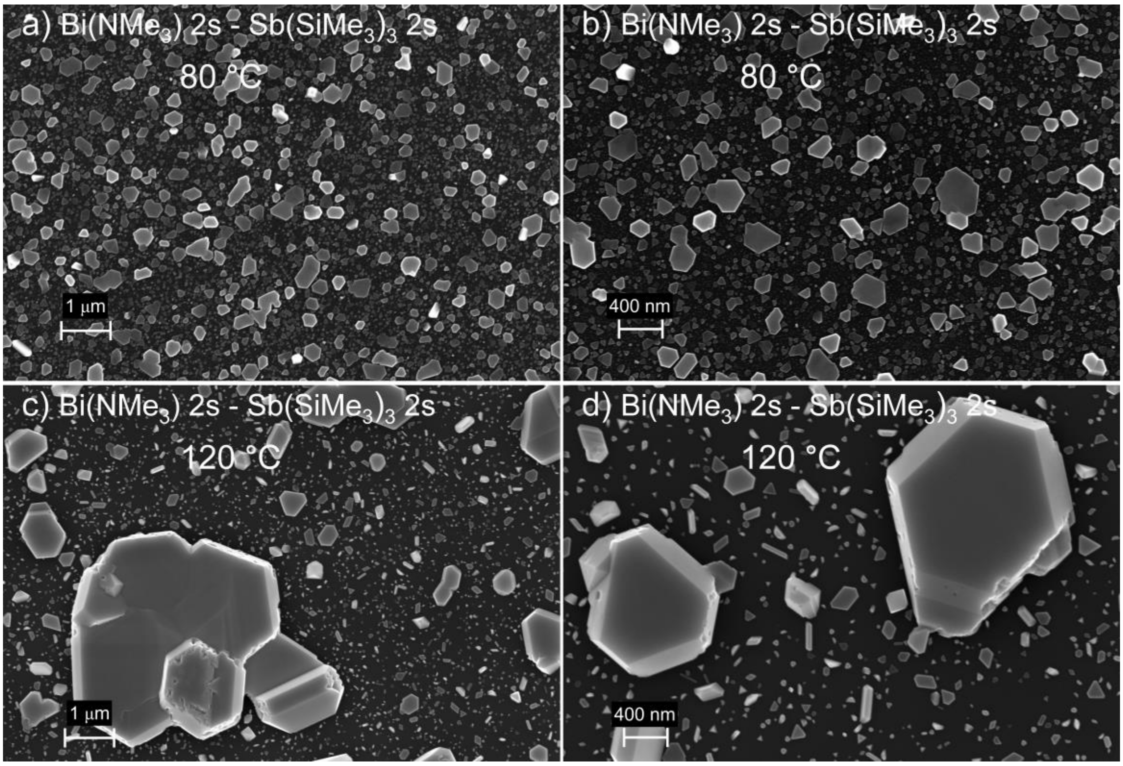


Figure S3. Top-view SEM Micrographs for 1000 ALD Cycles by varying the deposition temperature at 80 ℃ and 120 ℃.

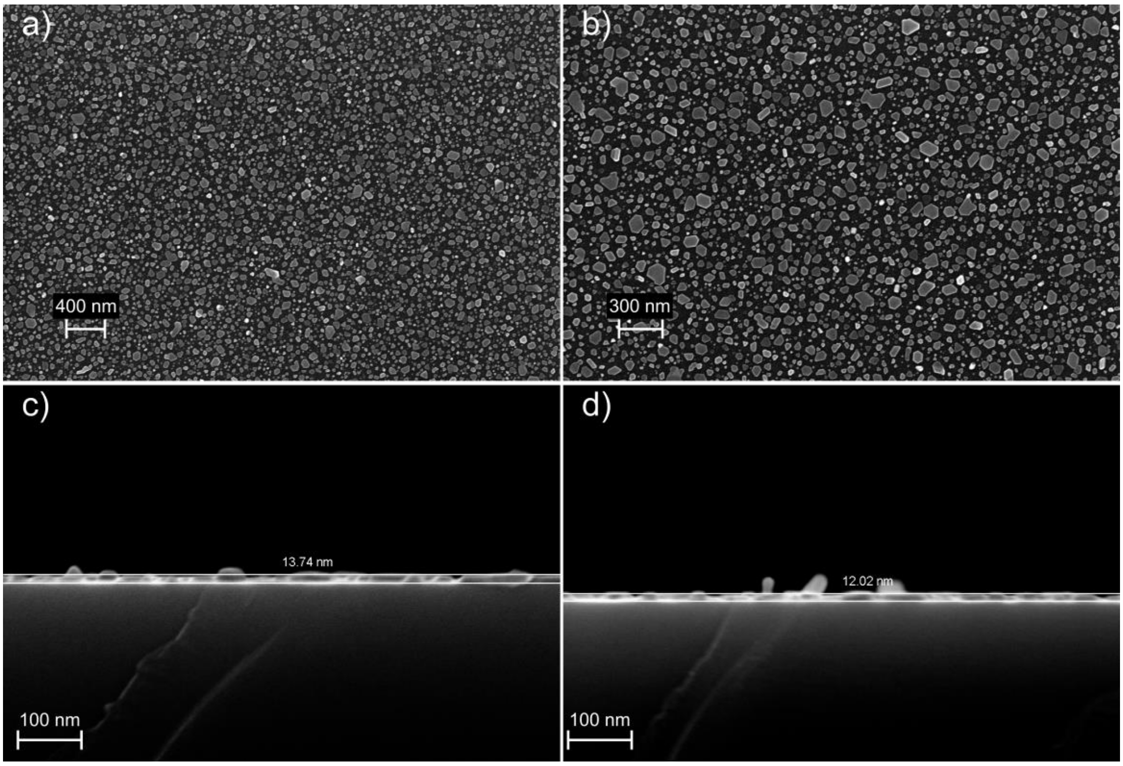


Figure S4. (a)-(b) Top-view and (c)-(d) cross-section SEM Micrographs for 100 ALD Cycles, surface coverage is around 20%.

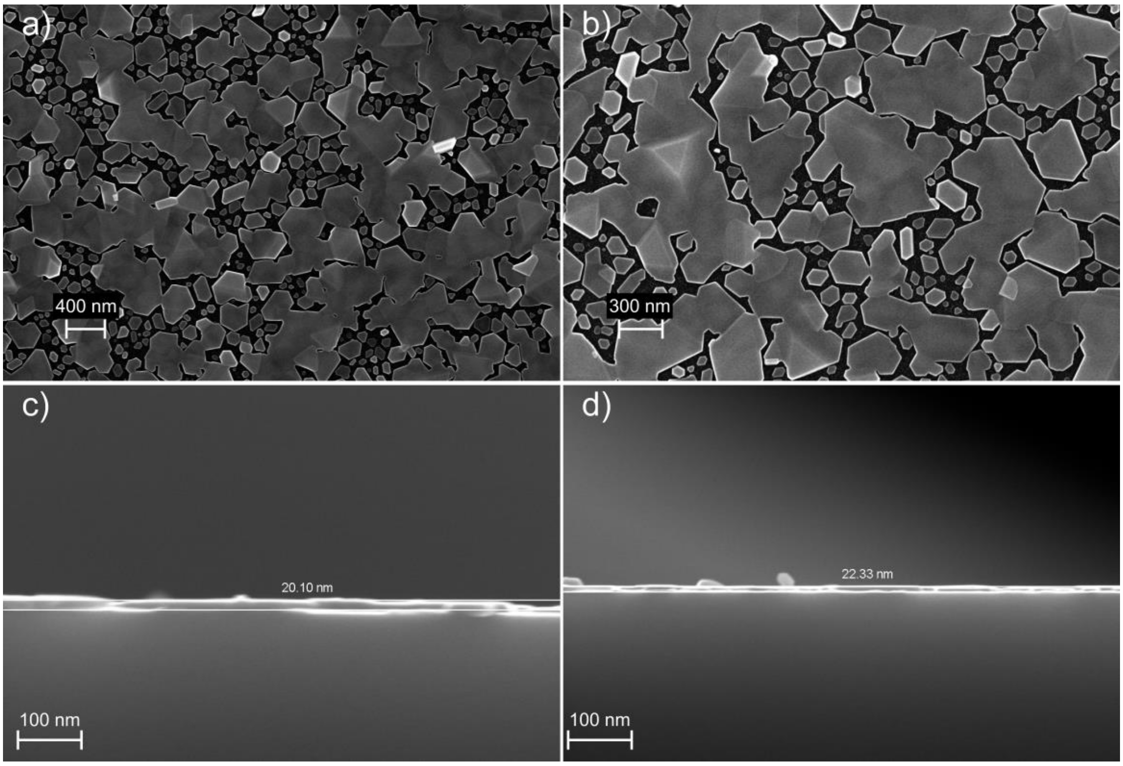


Figure S5. (a)-(b) Top-view and (c)-(d) cross-section SEM Micrographs for 500 ALD Cycles, surface coverage is around 50%.

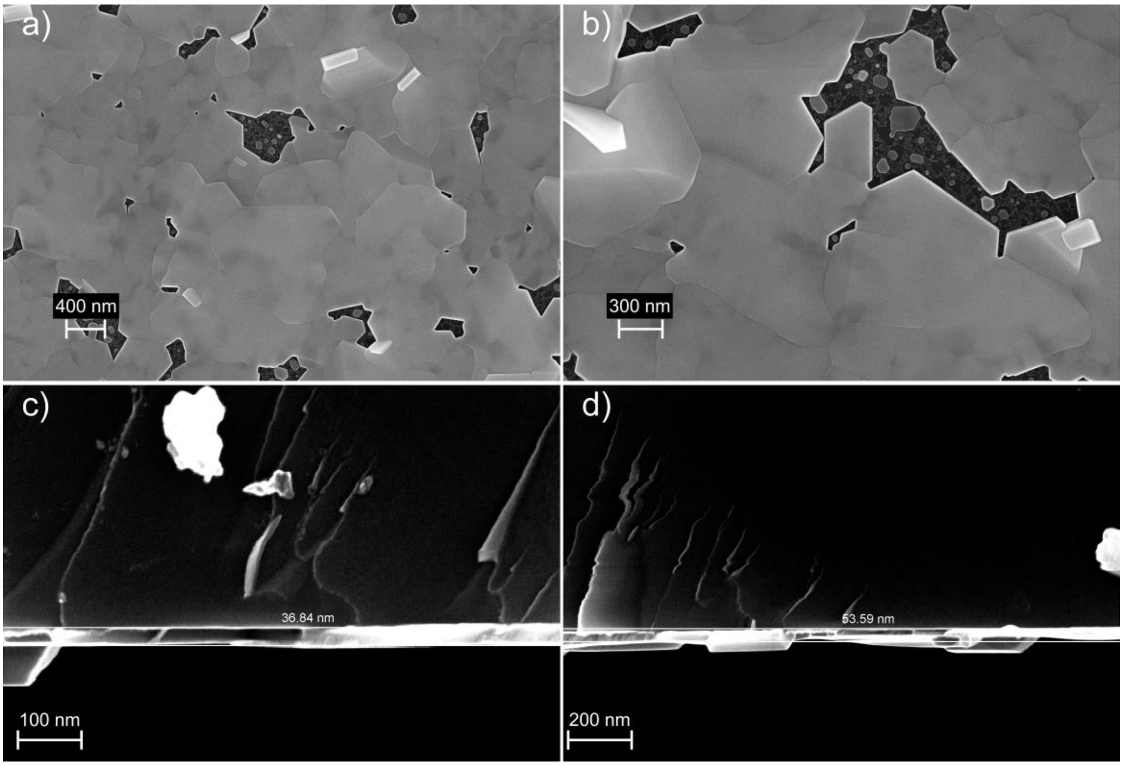


Figure S6. (a)-(b) Top-view and (c)-(d) cross-section SEM Micrographs for 1000 ALD Cycles, surface coverage is around 80%.

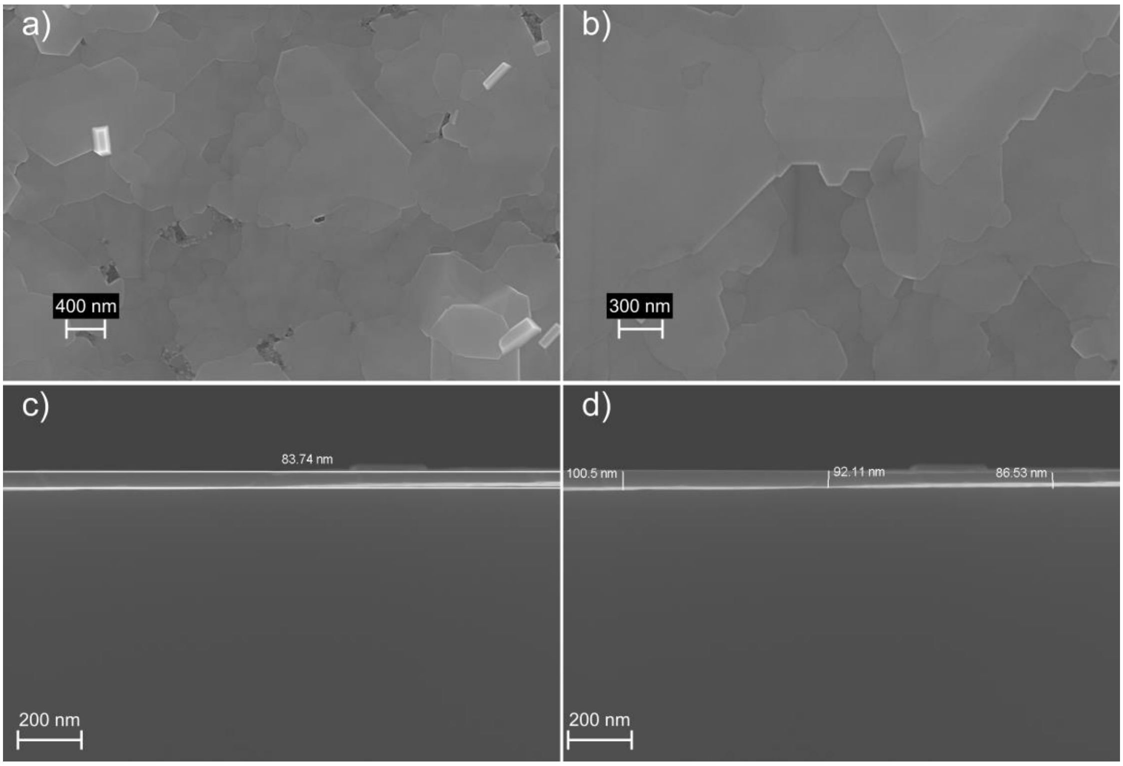


Figure S7. (a)-(b) Top-view and (c)-(d) cross-section SEM Micrographs for 2500 ALD Cycles, surface coverage is around 100%.

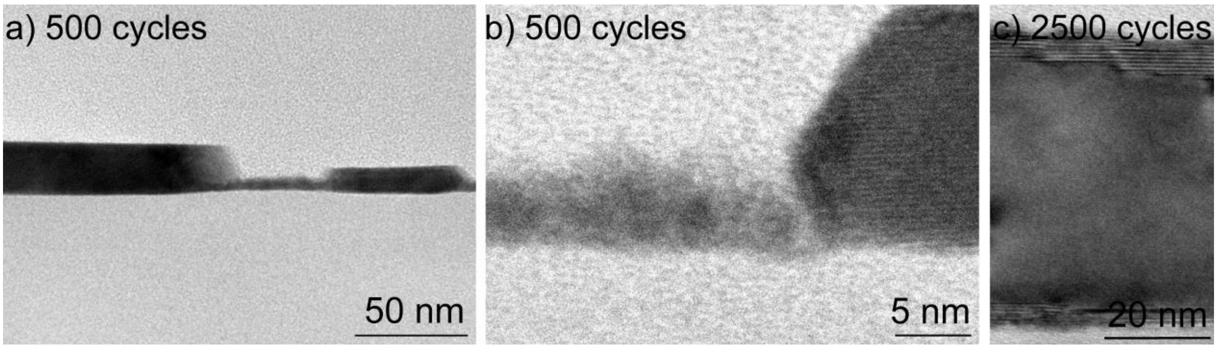


Figure S8. Cross-section TEM Micrographs for the (a)-(b) 500 and (c) 2500 cycles.

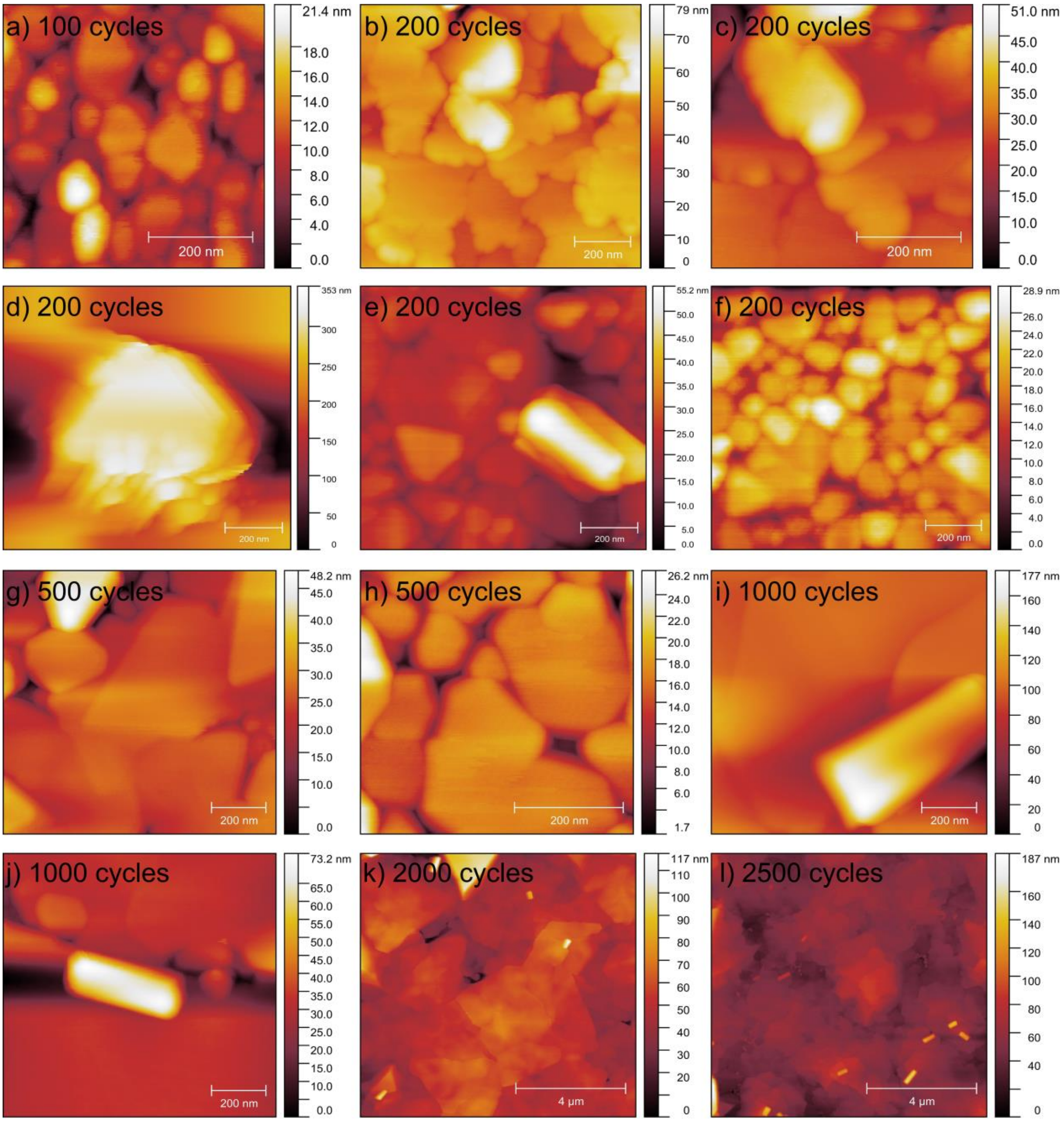


Figure S9. AFM images of the topography of Bi films of different cycles.

# 3 References

[1] M. Rusek, T. Komossa, G. Bendt, and S. Schulz, "Bismuth amides as promising ALD precursors for Bi2Te3 films," *J. Cryst. Growth*, vol. 470, pp. 128–134, Jul. 2017, doi: 10.1016/J.JCRYSGRO.2017.04.019.

[2] E. Amberger and R. W. Salazar G, "Mixed organometallic compounds of group V I. Synthesis of tris(trimethyl-group-IV)stibines," *J. Organomet. Chem.*, vol. 8, no. 1, pp. 111–114, Apr. 1967, doi: 10.1016/S0022-328X(00)84710-5.

[3] S. Schulz, "Organoaluminum Complexes with Bonds to s-Block, p-Block, d-Block, and f-

Block Metal Centers," pp. 59–90, 2012, doi: 10.1007/3418_2012_33.

[4] C. M. Evans, S. L. Castro, J. J. Worman, and R. P. Raffaelle, "Synthesis and use of tris(trimethylsilyl)antimony for the preparation of InSb quantum dots," *Chem. Mater.*, vol. 20, no. 18, pp. 5727–5730, Sep. 2008, doi: 10.1021/CM702856V/ASSET/IMAGES/LARGE/CM-2007-02856V_0001.JPEG.

[5] "Synthetic Methods of Organometallic and Inorganic Chemistry," *Synth. Methods Organomet. Inorg. Chem.*, Jul. 1996, doi: 10.1055/B-0035-108134.

[6] F. Neese, F. Wennmohs, U. Becker, and C. Riplinger, "The ORCA quantum chemistry program package," *J. Chem. Phys.*, vol. 152, no. 22, Jun. 2020, doi: 10.1063/5.0004608.

[7] F. Neese, "The ORCA program system," *WIREs Comput. Mol. Sci.*, vol. 2, no. 1, pp. 73–78, Jan. 2012, doi: 10.1002/wcms.81.

[8] F. Neese, "Software update: the ORCA program system, version 4.0," *WIREs Comput. Mol. Sci.*, vol. 8, no. 1, Jan. 2018, doi: 10.1002/wcms.1327.

[9] F. Neese, "Software update: The ORCA program system—Version 5.0," *WIREs Comput. Mol. Sci.*, vol. 12, no. 5, Sep. 2022, doi: 10.1002/wcms.1606.

[10] J. P. Perdew, K. Burke, and M. Ernzerhof, "Generalized Gradient Approximation Made Simple [Phys. Rev. Lett. 77, 3865 (1996)]," *Phys. Rev. Lett.*, vol. 78, no. 7, pp. 1396–1396, Feb. 1997, doi: 10.1103/PhysRevLett.78.1396.

[11] J. P. Perdew, K. Burke, and M. Ernzerhof, "Generalized Gradient Approximation Made Simple," *Phys. Rev. Lett.*, vol. 77, no. 18, pp. 3865–3868, Oct. 1996, doi: 10.1103/PhysRevLett.77.3865.

[12] C. Lee, W. Yang, and R. G. Parr, "Development of the Colle-Salvetti correlation-energy formula into a functional of the electron density," *Phys. Rev. B*, vol. 37, no. 2, pp. 785–789, Jan. 1988, doi: 10.1103/PhysRevB.37.785.

[13] A. D. Becke, "Density-functional thermochemistry. III. The role of exact exchange," *J. Chem. Phys.*, vol. 98, no. 7, pp. 5648–5652, Apr. 1993, doi: 10.1063/1.464913.

[14] S. H. Vosko, L. Wilk, and M. Nusair, "Accurate spin-dependent electron liquid correlation energies for local spin density calculations: a critical analysis," *Can. J. Phys.*, vol. 58, no. 8, pp. 1200–1211, Aug. 1980, doi: 10.1139/p80-159.

[15] P. J. Stephens, F. J. Devlin, C. F. Chabalowski, and M. J. Frisch, "Ab Initio Calculation of Vibrational Absorption and Circular Dichroism Spectra Using Density Functional Force Fields," *J. Phys. Chem.*, vol. 98, no. 45, pp. 11623–11627, Nov. 1994, doi:

10.1021/j100096a001.

[16] E. Caldeweyher *et al.*, “A generally applicable atomic-charge dependent London dispersion correction,” *J. Chem. Phys.*, vol. 150, no. 15, Apr. 2019, doi: 10.1063/1.5090222.

[17] E. Caldeweyher, J.-M. Mewes, S. Ehlert, and S. Grimme, “Extension and evaluation of the D4 London-dispersion model for periodic systems,” *Phys. Chem. Chem. Phys.*, vol. 22, no. 16, pp. 8499–8512, 2020, doi: 10.1039/D0CP00502A.

[18] E. Caldeweyher, C. Bannwarth, and S. Grimme, “Extension of the D3 dispersion coefficient model,” *J. Chem. Phys.*, vol. 147, no. 3, Jul. 2017, doi: 10.1063/1.4993215.

[19] F. Weigend and R. Ahlrichs, “Balanced basis sets of split valence, triple zeta valence and quadruple zeta valence quality for H to Rn: Design and assessment of accuracy,” *Phys. Chem. Chem. Phys.*, vol. 7, no. 18, p. 3297, 2005, doi: 10.1039/b508541a.

[20] F. Weigend, “Accurate Coulomb-fitting basis sets for H to Rn,” *Phys. Chem. Chem. Phys.*, vol. 8, no. 9, p. 1057, 2006, doi: 10.1039/b515623h.

[21] G. Bistoni, C. Riplinger, Y. Minenkov, L. Cavallo, A. A. Auer, and F. Neese, “Treating Subvalence Correlation Effects in Domain Based Pair Natural Orbital Coupled Cluster Calculations: An Out-of-the-Box Approach,” *J. Chem. Theory Comput.*, vol. 13, no. 7, pp. 3220–3227, Jul. 2017, doi: 10.1021/acs.jctc.7b00352.

[22] C. Riplinger, P. Pinski, U. Becker, E. F. Valeev, and F. Neese, “Sparse maps—A systematic infrastructure for reduced-scaling electronic structure methods. II. Linear scaling domain based pair natural orbital coupled cluster theory,” *J. Chem. Phys.*, vol. 144, no. 2, Jan. 2016, doi: 10.1063/1.4939030.

[23] C. Riplinger, B. Sandhoefer, A. Hansen, and F. Neese, “Natural triple excitations in local coupled cluster calculations with pair natural orbitals,” *J. Chem. Phys.*, vol. 139, no. 13, Oct. 2013, doi: 10.1063/1.4821834.

[24] F. Neese, A. Hansen, and D. G. Liakos, “Efficient and accurate approximations to the local coupled cluster singles doubles method using a truncated pair natural orbital basis,” *J. Chem. Phys.*, vol. 131, no. 6, Aug. 2009, doi: 10.1063/1.3173827.

[25] F. Neese, F. Wennmohs, and A. Hansen, “Efficient and accurate local approximations to coupled-electron pair approaches: An attempt to revive the pair natural orbital method,” *J. Chem. Phys.*, vol. 130, no. 11, Mar. 2009, doi: 10.1063/1.3086717.

[26] C. Riplinger and F. Neese, “An efficient and near linear scaling pair natural orbital based local coupled cluster method,” *J. Chem. Phys.*, vol. 138, no. 3, Jan. 2013, doi: 10.1063/1.4773581.

[27] D. G. Truhlar, “Basis-set extrapolation,” *Chem. Phys. Lett.*, vol. 294, no. 1–3, pp. 45–48, Sep. 1998, doi: 10.1016/S0009-2614(98)00866-5.

[28] F. Neese and E. F. Valeev, “Revisiting the Atomic Natural Orbital Approach for Basis Sets: Robust Systematic Basis Sets for Explicitly Correlated and Conventional Correlated *ab initio* Methods?,” *J. Chem. Theory Comput.*, vol. 7, no. 1, pp. 33–43, Jan. 2011, doi: 10.1021/ct100396y.

[29] F. Neese, F. Wennmohs, A. Hansen, and U. Becker, “Efficient, approximate and parallel Hartree–Fock and hybrid DFT calculations. A ‘chain-of-spheres’ algorithm for the Hartree–Fock exchange,” *Chem. Phys.*, vol. 356, no. 1–3, pp. 98–109, Feb. 2009, doi: 10.1016/j.chemphys.2008.10.036.

[30] D. Bykov *et al.*, “Efficient implementation of the analytic second derivatives of Hartree–Fock and hybrid DFT energies: a detailed analysis of different approximations,” *Mol. Phys.*, vol. 113, no. 13–14, pp. 1961–1977, Jul. 2015, doi: 10.1080/00268976.2015.1025114.

[31] B. Helmich-Paris, B. de Souza, F. Neese, and R. Izsák, “An improved chain of spheres for exchange algorithm,” *J. Chem. Phys.*, vol. 155, no. 10, Sep. 2021, doi: 10.1063/5.0058766.

[32] F. Neese, “An improvement of the resolution of the identity approximation for the formation of the Coulomb matrix,” *J. Comput. Chem.*, vol. 24, no. 14, pp. 1740–1747, Nov. 2003, doi: 10.1002/jcc.10318.
